# Assessing Psychological Disorders in Turkish Adolescents with Transfusion-Dependent Thalassemia

**DOI:** 10.3390/children11070837

**Published:** 2024-07-09

**Authors:** Aylin Yetim Şahin, Ibrahim Kandemir, Hüseyin Dağ, Emine Türkkan, Melike Tuğrul Aksakal, Memduh Sahin, Firdevs Baş, Zeynep Karakaş

**Affiliations:** 1 Department of Pediatrics, Division of Adolescent Medicine, Faculty of Medicine, Istanbul University, 34452 Istanbul, Turkey; aylin.yetim@istanbul.edu.tr (A.Y.Ş.); mzeynep@istanbul.edu.tr (M.T.A.); firdevsb@istanbul.edu.tr (F.B.); 2 Department of Pediatrics, Faculty of Medicine, Biruni University, 34452 Istanbul, Turkey; 3 Department of Pediatrics, University of Health Sciences, Istanbul Prof. Dr. Cemil Tascioglu City Hospital, 34452 Istanbul, Turkey; huseyin.dag@sbu.edu.tr; 4 Department of Pediatric Hematology and Oncology, University of Health Sciences, Istanbul Prof. Dr. Cemil Tascioglu City Hospital, 34452 Istanbul, Turkey; emine.turkkan1@saglik.gov.tr; 5 Department of Internal Medicine, University of Health Sciences, Istanbul Basaksehir Cam and Sakura Hospital, 34452 Istanbul, Turkey; memduh.sahin@sbu.edu.tr; 6 Department of Pediatric Hematology and Oncology, Faculty of Medicine, Istanbul University, 34452 Istanbul, Turkey; zkarakas@istanbul.edu.tr

**Keywords:** transfusion-dependent thalassemia, beta-thalassemia major, RCADS, depression, anxiety

## Abstract

We investigated depression and anxiety levels and related psychological disorders in adolescents with transfusion-dependent thalassemia (TDT) in this study. The study was conducted in two pediatric hematology outpatient clinics and included adolescents with TDT (14.8 ± 2.4 years, n = 40) in the study and compared them with the healthy age-matched control group (14.3 ± 2.3 years, n = 62). The Turkish version of the Revised Child Anxiety and Depression Scale (RCADS) was used to determine depression, anxiety, and related psychologic disorders (obsession, panic disorder, social phobia). Depression, anxiety, obsession, panic disorder, and social phobia scores were significantly higher in the patient group compared with the control (all *p* < 0.05). Ferritin levels were positively correlated with total depression, general anxiety, separation anxiety, and social phobia scores, but transfusion frequency and young age were the confounding factors. Patients in early adolescence and those who require more frequent blood transfusions are at higher risk of developing psychological disorders; routine screening for mood disorders should be warranted. Serum ferritin level may be a good warning indicator for early recognition of psychologic disorders in TDT patients.

## 1. Introduction

Beta-thalassemia is one of the hematological diseases that cause anemia defined in three groups: minor (carrier), intermedia (partial transfusion-dependent), and major (severe transfusion-dependent) [1]. Patients with transfusion-dependent thalassemia (TDT) need to undergo routine red-cell pack transfusions; however, this therapy may contribute to iron storage in the heart, liver, kidney, pancreas, and even in the pituitary gland, leading to chronic heart failure, chronic liver disease, diabetes, hypogonadism, and short stature [2].

Children and adolescents with chronic illnesses may have intense stress. There are studies reporting that children with chronic conditions may suffer depression and anxiety, which need psychological support [3]. Illnesses and hospitalizations might harm mental health in children, which might extend to adulthood [4]. Likewise, depression and anxiety rates are higher in patients with beta-thalassemia major, which indicates the need for psychiatric follow-up [5]. Higher depression and anxiety were reported even in patients with beta-thalassemia minor [6], which should raise mental health concerns in patients with thalassemia. Children diagnosed with hematological disorders, especially TDT or illnesses with painful episodes like sickle cell disease, should be under psychological follow-up to prevent or treat mental disorders [7]. Depressive symptom screening is vital in TDT patients to improve psychological and emotional health [8].

In the literature, there is evidence of depression and anxiety risk in TDT adolescents. However, there is limited information about related psychological disorders, like obsession, panic disorder, social phobia, and affecting factors. In this study, we aimed to determine depression and anxiety levels and related psychologic disorders in adolescents with TDT, as well as to research the effects of gender, age, transfusion frequency, and serum ferritin levels on them.

## 2. Materials and Methods

The study was conducted in the Istanbul University Faculty of Medicine and Prof Dr. Cemil Taşçıoğlu City Hospital pediatric hematology outpatient clinics between February and August 2018. 

### 2.1. Participants

The adolescents with TDT (transfusion once, twice, or three times a month) aged between 10 and 18 years old with a mean age of 14.8 ± 2.4 years and literate were invited to the study. Patients with any chronic illness (cancer, cardiac, pulmonary, neurologic, endocrinologic, gastrointestinal, hematologic, immunity, locomotor system, etc.), undergoing acute or chronic treatment (immune-suppressive, steroids, antithrombotic treatments, etc.), patients with a known psychological disease, autism spectrum disorder or intellectual disabilities, under psychiatric support or with an acute illness that may impact the scores were excluded. The age-matched control group consisted of adolescents who came to the adolescent outpatient clinic for routine check-ups. The control group consisted of healthy peers having no chronic disease, psychological disease, autism spectrum disorder, or intellectual disability, and the participants were healthy at the time they filled out the RCADS scale. A pediatrician taught the children how to complete the test, and the children performed the test in another place outside the hospital.

Participants read and filled out the RCADS scale in a separate room located outside the clinic. All the adolescents and parents were informed and gave written consent to participate in this study. The study is conducted with adherence to the Declaration of Helsinki, and the study permission was obtained from the Istanbul University Faculty of Medicine Ethical Committee (2018/192). 

### 2.2. Instruments 

The Revised Child Anxiety and Depression Scale (RCADS) was introduced as a tool for the assessment of pediatric anxiety and depression [9], of which validity was confirmed with a meta-analysis as a cross-cultural scale [10]. The validity of the RCADS was also confirmed in Turkish children [11].

The RCADS is a child self-report scale consisting of 47 items in total with six subscales, which are described as general anxiety (6 questions), separation anxiety (7 questions), panic disorder (9 questions), social phobia (9 questions), obsession (6 questions), and depression (10 questions). RCADS is a good psychometric scale and has demonstrated good reliability and validity in all sample groups [9]. Each answer has four choices: never, sometimes, often, and always (counted as 0, 1, 2, and 3 points, respectively). Scores above 70 points were defined as increased scores (at risk for the relevant scale) [9].

### 2.3. Statistical Analysis

Data were presented as mean ± standard deviation, median (interquartile range), and n (%). We used the Shapiro–Wilk test to assess normality and Spearman’s test to assess the correlation between two groups with non-normal data. To compare factors in multivariate analysis, a linear regression test (LR) was applied in which model the assumptions (Durbin–Watson test for autocorrelation, variance inflating factor for collinearity, and Shapiro–Wilk test for normality) are met, otherwise we used generalized linear model (GLM). The backward elimination method was used to reveal significant confounders between dummies in multivariate analysis with multiple factors. The correlation strength of the models is given with R^2^. A type-1 error *p* < 0.05 was considered of statistical significance. Bayesian Kendall’s tau test was applied to assess the null hypothesis, with a transformation to correct the skewness of the data if needed. All the calculations were performed by JAMOVI 2.3.18 with *gamlj* and *jsq* extensions.

## 3. Results

A total of 40 adolescents with TDT and 62 healthy peers were included in the study (mean ages: 14.8 ± 2.4 and 14.3 ± 2.3 years old, respectively; range: 10−18). The groups were similar regarding age (*p* = 0.149, Mann–Whitney U test) and gender (*p* = 0.368, chi-square test) (Table 1). 

The subscale results regarding gender and groups are presented in Table 2. 

**Figure 1:**
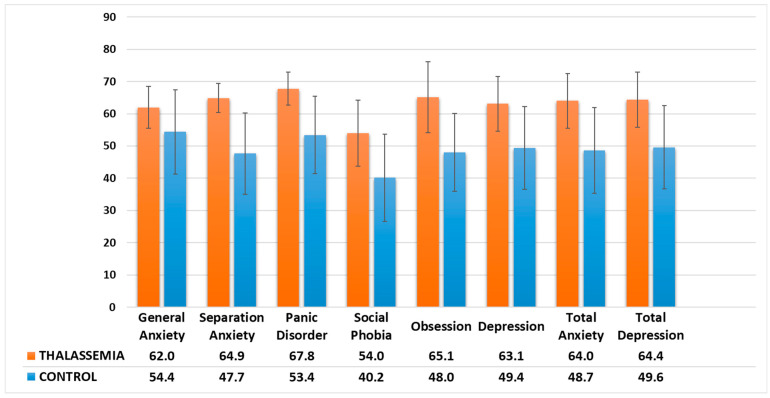
Comparison of the RCADS subscale scores between the adolescents with TDT and the healthy control group.

All subscale scores were higher in the TDT group (Figure 1). The comparison of the RCADS subscale scores and the percentage of increased scores (>70 points) are presented in Table 3.

**Figure 2:**
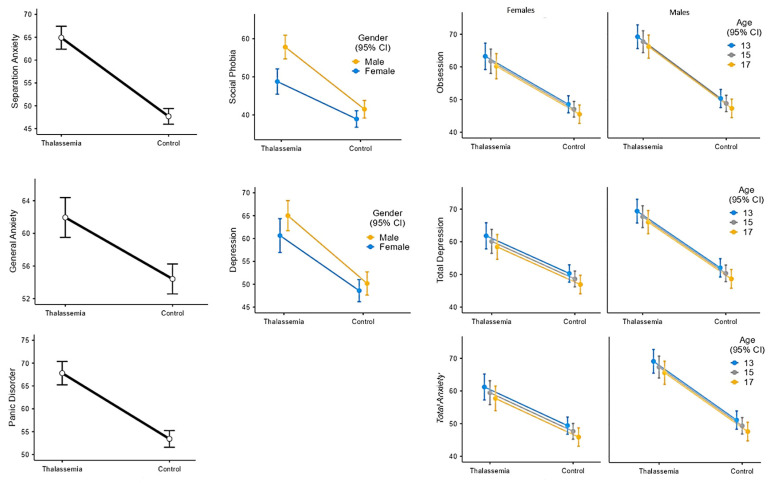
The RCADS subscales regarding confounders (group, age, and gender).

Multivariate regression models with the patient and the control groups were built and subjected to gender and age as covariates to reveal confounders. The statistically significant confounders were analyzed using the backward elimination method. The separation anxiety, general anxiety, and panic disorder scales were not affected by age and gender. All subscale scores were higher in the patient group compared with the controls. The separation anxiety score was higher by 17.2 points (95% CI: 14.2−20.3, GLM, R^2^: 0.453), the general anxiety score was higher by 7.5 points (95% CI: 4.1−11, LR, R^2^: 0.156), panic disorder points was higher by 14.4 points (95% CI: 11.3−17.5, GLM, R^2^: 0.385) in TDT group compared with the control (Figure 2).

The social phobia and depression subscale scores were affected by gender. Social phobia score was higher by 13.1 points (95% CI: 10.3−15.9) in the TDT group and 5.8 points (95% CI: 3.0−8.6) in boys (GLM, R^2^: 0.302). The depression scale score was higher by 13.4 points (95% CI: 10.4−16.5) in the TDT group and 3.0 points (95% CI: −0.1−6.0) in boys (GLM, R^2^: 0.321) (Figure 2).

The obsession, total depression, and total anxiety scales were affected by both gender and age. Obsession scale score was higher by 16.8 points (95% CI: 13.8−19.9) in the TDT group, 3.9 points (95% CI: 0.8−6.9) in boys, and increased by 0.8 points (95% CI: 0.1−1.4) with per age decrease in years (GLM, R^2^: 0.382). Total depression score was higher by 14.4 points (95% CI: 11.4−17.5) in the TDT group, 4.6 points (95% CI: 1.6−7.7) in boys, and increased by 0.8 points (95% CI: 0.2−1.5) with per decrease in age (GLM, R^2^: 0.383). Total anxiety score was higher by 14.9 points (95% CI: 11.9−18.0) in the TDT group, 4.9 points (95% CI: 1.9−7.9) in boys, and increased by 0.9 points (95% CI: 0.2−1.5) with per age decrease in years (GLM, R^2^: 0.393) (Figure 2).

All the scores of the subscales were higher in the TDT group compared with controls. The scores of the obsession, total depression, total anxiety, social phobia, and depression subscales were higher in boys than in girls. Age was a protective factor for obsession, total depression, and total anxiety scale scores. 

### Assessing the Utmost Vulnerable Subpopulation among TDT Patients

**Figure 3:**
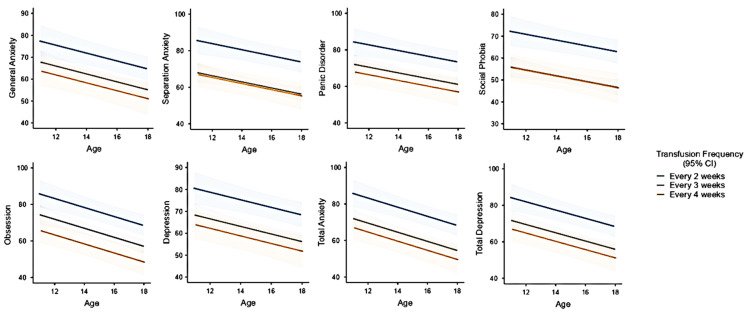
The associations of transfusion frequency (one, two, or three transfusions per month) with RCADS subscale scores. The lines refer to transfusion frequency and 95% CI (from top to below: every two weeks, every three weeks, and every four weeks, respectively).

The utmost vulnerable subpopulation within the TDT group was evaluated by assessing transfusion frequency, serum ferritin level, age, and gender in multivariate models. The backward elimination method was performed to reveal statistically significant confounding factors. Transfusion frequency and age were the utmost factors in the subscales in the TDT patients. The graphics and the estimated marginal mean table of the mentioned multivariate models are presented in Figure 3 and Table 4.

As hypothesized that ferritin level has any relation with subscale scores (H_1_), Bayesian statistics is applied, and the null hypothesis was defined as ferritin has no impact on elevated scores (H_0_). The obsession and depression scales resulted in anecdotal evidence, and panic disorder and total anxiety scales moderate evidence for the null hypothesis. The total depression scale resulted in anecdotal evidence; general anxiety, separation anxiety, and social phobia resulted in moderate evidence for the H_1_ hypothesis (Table 5).

## 4. Discussion

Chronic illnesses may cause psychological problems in childhood [3,12], and it is known that adolescents with anxiety or depression are more likely to have risky behaviors such as substance use, early pregnancy, suicide, and low educational or psychosocial functioning [8]. Moreover, depression and anxiety problems in childhood might extend to adulthood [13]. Hence, it is crucial to reveal vulnerable populations in childhood and initiate treatment on time. Thalassemia is one of the diseases that cause various complications, including physical, social, and emotional [1]. Depression and anxiety are expected problems in these patients [7,8]. Adolescence is a more sensitive period than the others, and the psychological problems related to thalassemia disease become even more obvious during this period. Youth with TDT should be followed more carefully, not only about depression and/or anxiety but also about other related psychological disorders. 

Beta-thalassemia major is a devastating illness that affects the education of school-aged children at a rate of 60% [14]. Diseases that negatively affect school attendance form the basis for depression and anxiety. Chronic diseases are associated with mood disorders, as Afifi et al. [3] reported that depression and anxiety were more prevalent in TDT patients compared with hemodialysis or malignancy patients. In comparison with sickle cell anemia, the majority of patients (89.3% for depression and 93.3% for anxiety) were below the pathologic threshold in TDT patients. They did not find age or gender effect on depression or anxiety among 10−17-year-old patients. They also did not find a significant effect of pain frequency in the multivariate models [7]. However, depression symptoms were reported in 60% of patients among TDT patients [15], whereas another study reported a depression rate of 19.8% in adults diagnosed with beta-thalassemia major [16]. Similar to our results, a meta-analysis conducted with beta-thalassemia major-diagnosed adult and children studies concluded that 42% of patients had depression [17]. Anxiety levels have also been recognized as higher in this patient group, especially among adolescents and young adults [17]. Adib-Hajbaghery et al. [18] presented that 58.4% of patients with TDT (32% of whom were adolescents) had mild to severe anxiety symptoms. This vulnerable group is also at risk in this respect in the later years of life, as a study reported that TDT patients have worse life quality and more depression and anxiety presence [19]. In line with our study results and the literature, it can be offered that every adolescent with TDT should undergo psychosocial evaluation in terms of depression and anxiety symptoms. 

Studies examining psychological problems other than depression and/or anxiety in TDT patients are limited. Besides depression, Messina et al. [20] reported that somatization and obsessive-compulsive disorder are also recognized more often in TDT patients. Separation anxiety is researched in only one study, which gives a rate of 4.8% [21]. In our study, elevated scores in the TDT group for obsession were 42.5%, 55% for panic disorder, 12,5% for social phobia, and 40% for separation anxiety. These rates suggest that adolescents with TDT are also at risk in terms of other related psychological problems and may need psychiatric evaluation in this regard.

Boys with severe diseases tend to have more mood disorders in the literature. A study conducted with leukemia/lymphoma patients in remission reported that boys tend to have more psychiatric diseases, such as anxiety (17%), depression (15%), separation anxiety (25%), obsessive-compulsive disorder (40%), and panic disorder (35%) [22]. Similarly, social phobia, depression, obsession, and total anxiety scores were higher in boys with TDT in this study. In fact, girls have more tendency to depression and anxiety in the normal population as publications are reporting higher depression and anxiety among 15−18-year-old students [23] and 12−17-year-old bullied girls [24]. But this tendency was reciprocated by boys in our TDT patients. Therefore, clinicians should be aware of the risk in this gender group. 

Younger age (<15 years old) and frequent transfusion (biweekly), but not gender, had the statistical effect on RCADS subscales scores. The patients in early adolescence resulted in higher RCADS scores in obsession, total depression, and total anxiety subscales. During early adolescence, rapid growth, development, and the onset of pubertal symptoms are observed. Failure to achieve expected growth in TDT patients in this age group, the emergence of problems such as delayed puberty, and, of course, intensive medical treatments may explain the frequency of psychological problems in early adolescence. Transfusion frequency was an influential factor in the literature; two studies reported similar results to ours, as transfusion frequency was positively correlated with the presence of depression in TDT patients [25,26]. However, it is not known if serum ferritin levels have an effect on mood disorders. In light of this, a study suggested that iron accumulation with blood transfusions may cause an immune-inflammatory response, and both inflammation and oxidative stress toxicity might cause depression episodes [26]. As we hypothesized that serum ferritin levels might affect depression and anxiety, the multivariate analysis revealed that serum ferritin levels did not reach statistical significance if subjected to transfusion frequency, age, and gender in the TDT group. Also, obsession, depression, panic disorder, and total anxiety scales resulted favoring null hypothesis, but general anxiety, separation anxiety, and social phobia scores resulted in moderate evidence, which means serum ferritin levels might have an impact on these subscales. In summary, transfusion frequency has a significant impact on the RCADS subscales, more than serum ferritin levels, but serum ferritin levels might have a slight effect on anxiety and social phobia disorders. The positive correlation found between ferritin level and psychological disorder scores may be due to frequent transfusion and, therefore, changes in iron storage in the body, including the brain. To reach a conclusion about this hypothesis, radiology-supported studies with large sample sizes on this issue are needed if ferritin or iron deposition in the brain has a negative effect on these psychological disorders.

This is the first study presenting data about obsession, panic disorder, social phobia, and separation anxiety, along with depression and anxiety in adolescents with TDT. However, the information about the socioeconomic status and education level of families was lacking in this study and was one of the limitations.

## 5. Conclusions

Adolescents with TDT have a higher risk of developing anxiety, depression, and associated psychological disorders, such as obsession, panic disorder, and social phobia, compared to healthy ones. Therefore, routine screening for mood disorders should be recommended in this patient group. Adolescents with TDT who need frequent transfusions (every two weeks) and ones in early adolescence (younger than 15) have the utmost risk for anxiety and depression; therefore, we suggest routine psychological follow-up in this subgroup. Clinicians should be aware of this psychosocial aspect, and a multidisciplinary therapeutic team approach (including adolescent medicine, social workers, psychologists, and psychiatrists) is essential. Developing a multidisciplinary approach to these patients in the early period and establishing a group culture by providing communication with similar patient groups may lead to psychosocial well-being in later ages. Further, ferritin level may be an indicator for early recognition of anxiety in TDT patients, but radiology-supported studies are needed in this field, as a longitudinal anxiety-status follow-up of a patient group might give precious data.

### Limitations

We did not record the parental employment status, family situation, households, or educational situation of the children in this study.

## Figures and Tables

**Table 1:** Descriptive status of the patient and the control group regarding age.

	TDT Group			Control Group		
	Girls	Boys	Full Sample	Girls	Boys	Full Sample
Age	15.4 ± 2.6 (11−18)	15.1 ± 2.9 (11−18)	15.2 ± 2.8 (11−18)	14.6 ± 2.1 (10−18)	14.8 ± 1.9 (11−18)	14.7 ± 2.0 (10−18)

Data are presented as mean ± sd (minimum–maximum).

**Table 2:** Subscale results of the patient and the control group regarding the group and gender.

	TDT Group		Control Group	
	Girls	Boys	Girls	Boys
General Anxiety	59.3 ± 11.7 (42−80)	63.9 ± 13.2 (44−80)	54.2 ± 4.4 (43−64)	54.7 ± 4.7 (41−65)
Separation Anxiety	61.2 ± 13.5 (42−80)	67.7 ± 12.3 (46−80)	47.5 ± 6.9 (41−66)	47.9 ± 6.1 (38−65)
Panic Disorder	63.2 ± 12.9 (41−80)	71.2 ± 12.7 (46−80)	54.1 ± 5.3 (48−78)	52.7 ± 4.8 (40−65)
Social Phobia	48.8 ± 11.9 (31−80)	57.8 ± 13.6 (35−80)	38.9 ± 10.5 (30−66)	41.5 ± 10.1 (30−66)
Obsession	61.7 ± 12.1 (43−80)	67.7 ± 11.8 (47−80)	47.2 ± 11.5 (38−77)	48.9 ± 10.6 (38−70)
Depression	60.6 ± 12.2 (41−80)	65.0 ± 13.2 (42−0)	48.6 ± 8.7 (41−73)	50.2 ± 8.2 (41−65)
Total Anxiety	59.4 ± 12.7 (40−80)	67.4 ± 13 (42−80)	47.9 ± 9.0 (40−71)	49.6 ± 8.1 (40−61)
Total Depression	60.1 ± 12.4 (42−80)	67.6 ± 12.6 (45−80)	48.8 ± 9.0 (41−73)	50.5 ± 8.3 (41−62)

Data are presented as mean ± sd (minimum–maximum).

**Table 3:** Comparison of the RCADS subscale scores and individuals with elevated scores (70 points) between the adolescents with TDT and the healthy control group.

	RCADS Subscale Scores Mean ± SD			RCADS Elevated Score Percentage (>70 Points)		
	Patient (n = 40)	Control (n = 62)	*p*	Patient (n = 40)	Control (n = 62)	*p*
General Anxiety	62.0 ± 12.6	54.4 ± 4.5	0.003 ^M^	25.0% (n = 10)	0%	<0.001 ^F^
Separation Anxiety	64.9 ± 13.1	47.7 ± 6.4	<0.001 ^S^	40.0% (n = 16)	0%	<0.001 ^C^
Panic Disorder	67.8 ± 13.2	53.4 ± 5.1	<0.001 ^M^	55.0% (n = 22)	1.6% (n = 1)	<0.001 ^F^
Social Phobia	54.0 ± 13.5	40.2 ± 10.3	<0.001 ^M^	12.5% (n = 5)	0%	0.008 ^F^
Obsession	65.1 ± 12.1	48.0 ± 11.0	<0.001 ^M^	42.5% (n = 17)	1.6% (n = 1)	<0.001 ^F^
Depression	63.1 ± 12.8	49.4 ± 8.5	<0.001 ^M^	32.5% (n = 13)	1.6% (n = 1)	<0.001 ^F^
Total Anxiety	64.0 ± 13.3	48.7 ± 8.5	<0.001 ^M^	32.5% (n = 13)	1.6% (n = 1)	<0.001 ^C^
Total Depression	64.4 ± 12.9	49.6 ± 8.6	<0.001 ^M^	35.0% (n = 14)	1.6% (n = 1)	<0.001 ^C^

Data are presented as mean ± sd for presentation. ^M^—Mann–Whitney U test, ^S^—Student’s *t*-test, ^F^—Fisher’s exact test, ^C^—chi-square test.

**Table 4:** Estimated marginal means of the subscales with transfusion frequency and age.

Transfusion Frequency	Age	General Anxiety	Separation Anxiety	Panic Disorder	Social Phobia	Obsession	Depression	Total Anxiety	Total Depression
Every 2 weeks	12	75 (65−84)	83 (74−92)	82 (72−95)	70 (60−79)	82 (75−90)	78 (69−87)	82 (73−91)	81 (72−90)
	15	70 (62−78)	79 (71−86)	78 (69−86)	66 (58−75)	76 (69−82)	73 (66−81)	75 (68−83)	75 (67−82)
	18	65 (57−74)	74 (66−82)	74 (65−82)	63 (55−72)	69 (62−75)	69 (60−77)	69 (61−77)	69 (61−77)
Every 3 weeks	12	65 (59−72)	66 (60−72)	70 (63−76)	54 (47−60)	71 (66−76)	66 (60−72)	68 (63−74)	69 (63−74)
	15	61 (56−65)	61 (57−66)	66 (61−71)	50 (46−55)	64 (60−68)	61 (57−66)	62 (57−66)	62 (58−67)
	18	56 (50−62)	57 (51−62)	62 (55−68)	47 (41−53)	57 (52−62)	56 (50−62)	55 (50−61)	56 (51−62)
Every 4 weeks	12	61 (51−71)	65 (55−74)	65 (55−76)	54 (44−64)	62 (54−70)	61 (52−71)	63 (54−72)	63 (54−73)
	15	56 (46−66)	60 (51−69)	61 (51−72)	50 (40−61)	55 (47−63)	56 (47−66)	56 (47−66)	57 (48−67)
	18	51 (40−63)	56 (45−66)	57 (45−70)	47 (35−59)	48 (39−57)	51 (40−63)	50 (39−61)	51 (40−62)

The linear regression model was applied. The results are given as rounded for presentation.

**Table 5:** Bayes factors of elevated scores of RCADS subscales regarding serum ferritin levels.

Scales	Bayesian Kendall’s Tau	BF_10_	Evidence
General Anxiety	0.263	3.294	Moderate (H_1_)
Separation Anxiety	0.292	6.291	Moderate (H_1_)
Panic Disorder	0.076	0.258	Moderate (H_0_)
Social Phobia	0.262	3.184	Moderate (H_1_)
Obsession	0.140	0.448	Anecdotal (H_0_)
Depression	0.158	0.554	Anecdotal (H_0_)
Total Anxiety	0.102	0.310	Moderate (H_0_)
Total Depression	0.205	1.1	Anecdotal (H_1_)

Note: ferritin levels are subjected to transformation for normalization. H_1_—alternative hypothesis; H_0_—null hypothesis. Stretched beta prior width assumed 1.

## Data Availability

The data presented in this study will be shared upon reasonable requests from the corresponding author but cannot be shared in public as authors have no permission granted by the ethical board.

## Abbreviations

TDT	Transfusion-dependent thalassemia
RCADS	Revised Child Anxiety and Depression Scale
IQR	Interquartile range
LR	Linear regression test
GLM	Generalized linear model
